# Thirty Years of Emergency Medicine in Romania—A Bridge Between the Behavior of Emergency Department Professionals and the Health System Management Strategy: A Survey Study

**DOI:** 10.3390/jcm14103316

**Published:** 2025-05-09

**Authors:** Adela Golea, Raluca M. Tat, Ștefan C. Vesa, Daniela Mitrofan, Cristian Boeriu, Luciana T. Rotaru, Diana C. Cimpoeșu, Silvia Nica, Alina Petrică, Monica Puticiu, Daniela Ionescu, Andrea Kazamer, Iris C. Mureșan

**Affiliations:** 1 Cluj-Napoca University County Emergency Clinical Hospital, 400006 Cluj-Napoca, Romania; adela.golea@umfcluj.ro; 2 Department 6 Surgery, Emergency Medicine Discipline, “Iuliu-Hațieganu” University of Medicine and Pharmacy, 400012 Cluj-Napoca, Romania; 3 Clinical Hospital of Infectious Diseases Cluj-Napoca, 400348 Cluj-Napoca, Romania; stefan.vesa@umfcluj.ro; 4 Department of Pharmacology, Toxicology and Clinical Pharmacy, “Iuliu-Hațieganu” University of Medicine and Pharmacy, 400012 Cluj-Napoca, Romania; 5 Cluj-Napoca Children’s Emergency Clinical Hospital, 400370 Cluj-Napoca, Romania; mitrodana@yahoo.com; 6 President of the Association for Interdisciplinary Study in the Field of Emergency Medicine, 400697 Cluj-Napoca, Romania; 7 Târgu Mureș County Emergency Clinical Hospital, 540136 Târgu Mureș, Romania; 8 Department of Clinical and Medical-Surgical Disciplines M3, “George Emil Palade” University of Medicine, Pharmacy, Sciences and Technology, 540142 Târgu-Mureș, Romania; 9 Craiova County Emergency Clinical Hospital, 200642 Craiova, Romania; luciana.rotaru@umfcv.ro; 10 Department 6 Surgery, Emergency Medicine Discipline, Craiova University of Medicine and Pharmacy, 200349 Craiova, Romania; 11 “St. Spiridon” County Emergency Clinical Hospital, 700111 Iași, Romania; carmen.cimpoesu@umfiasi.ro; 12 Emergency Department, “Grigore T. Popa” University of Medicine and Pharmacy Iași, 700115 Iași, Romania; 13 Bucharest Emergency University Hospital, 050098 Bucharest, Romania; silvia.nica@umfcd.ro; 14 Clinical Department 4, “Carol Davila” University of Medicine and Pharmacy Bucharest, 020021 Bucharest, Romania; 15 “Pius Brînzeu” County Emergency Clinical Hospital, 300723 Timișoara, Romania; alina.petrica@umft.ro; 16 Department 9 Surgery 1, “Victor Babeș” University of Medicine and Pharmacy, 300041 Timișoara, Romania; 17 Arad County Emergency Clinical Hospital, 310158 Arad, Romania; puticiu.monica@uvvg.ro; 18 Faculty of Medicine, Western University “Vasile Goldiș”, 310025 Arad, Romania; 19 Regional Institute of Gastroenterology and Hepatology “Professor Doctor Octavian Fodor”, 400162 Cluj-Napoca, Romania; daniela_ionescu@umfcluj.ro; 20 Department 6 Surgery, Discipline Anesthesia and Intensive Therapy 1, “Iuliu-Hațieganu” University of Medicine and Pharmacy, 400012 Cluj-Napoca, Romania; 21 CREST Association, 440069 Satu Mare, Romania; a.kazamer@crest.ro; 22 Sibiu County Emergency Clinical Hospital, 550245 Sibiu, Romania; iris.muresan@ulbsibiu.ro; 23 Department of Dental Medicine and Nursing, Faculty of Medicine, “Lucian Blaga” University Sibiu, 550024 Sibiu, Romania

**Keywords:** emergency medicine, risk management, human resources, patient, malpractice

## Abstract

**Background/Objectives:** Over the past three decades, emergency medicine in Romania has evolved from a developing specialty into a cornerstone of the national healthcare system. As we reflect on these 30 years, it becomes evident that the lessons learned and the systems developed form a vital foundation for the future. This study aims to explore how the accumulated experience can guide us toward building a more resilient emergency medical system, one that prioritizes quality, ensures patient and provider safety, and embraces modern principles of healthcare management. The objectives of this study were to explore the long-term perspectives of physicians and nurses working in emergency departments (EDs), to determine the triggering factors that may lead to abandoning the specialty, and to identify of malpractice risks arising from doctor–patient interaction. **Methods:** This study employed an observational design and utilized an opinion questionnaire to assess the participants’ perspectives. Qualitative data were presented as frequencies and percentages. Quantitative data were expressed as means and standard deviations after verifying normal distribution with the Shapiro–Wilk test. Comparisons between groups for qualitative variables were conducted using the chi-square test. For comparisons of quantitative variables between two groups, Student’s *t*-test was employed following confirmation of homogeneity of variances with Levene’s test. A *p*-value of < 0.05 was considered statistically significant. **Results:** Out of 1228 estimated responders, 641 completed the questionnaire. A total of 577 of participants met the inclusion criteria: 256 (44.4%) nurses and 321 (55.6%) doctors, with an average age of the responders of 40.06 years. Nurses reported the highest level of managerial support (83.2%, *p* < 0.001). EPs had the highest rate of non-participation in working groups for procedures/protocols/guidelines (49.5%, *p* < 0.001). Intensive care unit medical doctors (ICU-MDs) and EPs were the main groups reporting a deficiency in employer-provided resources to manage conflict situations (63.7%, 61.7%; *p* <0.001). EPs (28%) reported practicing defensive medicine (inadequate educational support, the absence of clear protocols). Workplace burnout was reported by the ICU-MDs and EPs responders (96.3%; 93.4%; *p* < 0.001), and 26% of EPs expressed interest in professional reorientation. **Conclusions:** This study highlights four strategic directions for rebuilding a resilient healthcare system focused on improving quality of care and safety: development of procedures/protocols, managerial reorganization, restoration of healthcare professionals’ trust through new strategies, and academic development.

## 1. Introduction

Emergency medicine is a novel specialty within the Romanian medical field, with the first emergency physicians (EPs) beginning their careers in 1997. According to local legislation (Ministry of Health Order 1706/2006), emergency departments (EDs) may employ not only emergency physicians but also doctors specializing in anesthesiology and intensive care (ICU-MDs) and related medical fields, as well as pediatricians, considering that emergency care addresses both pediatric and adult patients [1].

While both challenging and stimulating, the field of emergency medicine is currently facing a shortage of medical personnel across all professional categories, including doctors and nurses, as well as auxiliary staff, social workers, and administrative personnel [2,3]. At the end of 2022, the Romanian College of Physicians estimated that approximately 1112 emergency physicians were actively working in emergency medical services, both in the pre-hospital sector and in hospital EDs [2,3], with over 500 doctor positions left unfilled (based on an estimated need of 2000 emergency medicine specialists), reflecting a current personnel deficit of about 30–35% [2,3].

University centers feature a higher percentage of emergency physicians, with 50% of young specialists choosing to work in university medical centers, as a result of which the shortages are even more pronounced in smaller towns [2,3]. Although emergency medicine is relatively attractive compared to other medical specialties, the field is experiencing an increasing rate of training abandonment during the early years of residency (an estimated rate of 10–15%), further worsening the supply–demand imbalance. The remaining active medical personnel must compensate for the human resource deficit, which is reflected in a heavy workload, high stress level, development of burnout [4,5], and, importantly, an increased risk of adverse events in patient care, with potential repercussions in terms of malpractice (during the 2023–2024 period, over 1700 malpractice cases were registered within the Romanian judicial system, some of which involved emergency and intensive care physicians) [6,7].

Over the past three decades, emergency medicine in Romania has evolved from a developing specialty into a cornerstone of the national healthcare system. This period has been marked by significant advances in clinical practice, organizational structures, and professional training. As we reflect on these 30 years, it becomes evident that the lessons learned and the systems developed form a vital foundation for the future. This study aims to explore how the accumulated experience can guide us toward building a more resilient emergency medical system, one that prioritizes quality, ensures patient and provider safety, and embraces modern principles of healthcare management.

The objectives of this study were as follows:

1. To identify the main motivational factors that influenced the choice of emergency medicine as a specialty;

2. To understand how professionals’ perceptions of the field have evolved over the years;

3. To identify the precipitating factors that may lead to the abandonment of the specialty and/or professional reorientation;

4. To identify the potential malpractice risks arising from doctor–patient interactions.

## 2. Materials and Methods

### 2.1. Study Protocol

During the 4th Edition of the Symposium on Emergency Medicine and Pediatric Intensive Care, held in Cluj-Napoca, Romania, in November 2023 under the auspices of the Association for Interdisciplinary Studies in the Field of Emergency Medicine (ASIDU), a topic was introduced for debate concerning potential responses to the reorientation of medical careers or specialties. At this symposium, an initial group of 114 healthcare professionals responded to an anonymous questionnaire distributed electronically via the platform provided by the organizing company. The responders included nurses and doctors working in pediatric wards, emergency departments (EDs), and intensive care units (ICUs). Participation in this roundtable was not contingent upon formal registration for the symposium. Completion of the questionnaire was considered as providing informed consent to participate in the study, as was explicitly stated to responders.

As a challenging and current topic, it aimed to outline guiding ideas for the future that focus on developing the employee–organization relationship in EDs and on ensuring the safety of both medical practice and healthcare workers in order to strengthen the healthcare system characterized by safety and efficient management. Following on this hypothesis, a multicenter study was proposed, involving medical personnel from the same professional categories across Romania’s university medical centers, specifically Cluj-Napoca, Târgu Mureș, Sibiu, Arad, Timișoara, Craiova, Iași, and Bucharest, conducted between 1 November 2023 and 31 January 2024.

This descriptive cross-sectional survey study was conducted using an anonymous online questionnaire (opinion poll). Completion of the questionnaire was considered equivalent to giving informed consent, a point communicated to the responders from the university centers via a letter of intent. For the implementation of this study via the voluntary and anonymous opinion survey method, ethical approval (8 April 2024) was obtained from the ethics committee of Cluj-Napoca Emergency County University Hospital under number 16276/2024.

### 2.2. Inclusion Criteria

The inclusion criteria for this study included senior physicians specializing in emergency medicine, senior physicians in anesthesiology and intensive care, senior pediatricians, and nurses working in the ED. The exclusion criteria included other categories of healthcare workers, resident doctors (junior physicians), and medical specialties not listed among the inclusion criteria.

In this article, the term “senior” was used to refer to physicians who are fully credentialed to practice independently in emergency medicine.

### 2.3. Data Colection

The questionnaire was distributed via the Cisco Systems, Inc., platform, West Tasman, San Jose, CA, USA (https://www.slido.com, accessed on 1 November 2023) during the period from 1 November 2023 to 31 January 2024. The questionnaire consisted of a total of 14 questions, 3 open-ended (questions 5, 11, and 12) and 11 closed-ended (Figure S1).

### 2.4. Statistical Analysis

Statistical analysis was performed using the MedCalc^®^ Statistical Software version 22.021 (MedCalc Software Ltd., Ostend, Belgium; https://www.medcalc.org, accessed on 2 August 2024). Qualitative data were presented as frequencies and percentages. Quantitative data were expressed as means and standard deviations after verifying normal distribution with the Shapiro–Wilk test. Comparisons between groups for qualitative variables were conducted using the chi-square test. For comparisons of quantitative variables between two groups, Student’s t-test was employed following confirmation of homogeneity of variances with Levene’s test. A *p*-value of < 0.05 was considered statistically significant.

## 3. Results

**Figure 1:**
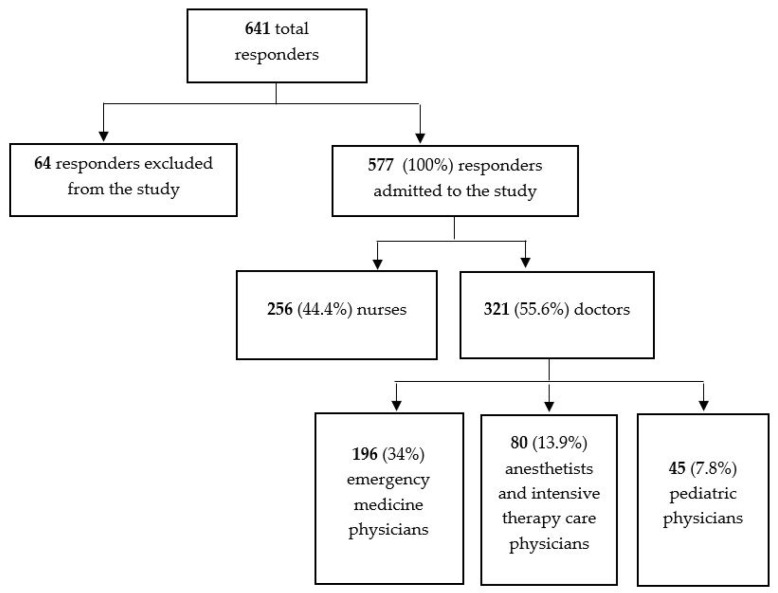
Diagram of the responders admitted to the study and their distribution by specialty.

This study targeted approximately 1228 responders (251 EPs, 137 ICU physicians, 80 pediatricians, and 760 emergency medicine nurses) from the hospitals of the University Centers in Cluj-Napoca, Târgu Mureș, Sibiu, Arad, Timișoara, Craiova, Iași, and Bucharest. Out of this estimated number, 641 (52.19%) responders completed the questionnaire. Out of a total of 641 completed questionnaires, 577 (90.01% of the responders) were included in the study. A total of 64 questionnaires were excluded because they did not meet the inclusion criteria (responders from other medical specialties or other professional categories such as radiology tehnicians, cleaners, stretcher bearers) or because they were improperly completed, making it impossible to determine professional affiliation. The average age of the responders was 40.06 years (standard deviation 9.58), with an average length of service of 14.11 years (standard deviation 9.86), primarily indicating under 20 years of professional experience. Among the responders included in the study, 424 (73.5%) were female (based on analysis of the first three questions). The distribution of responders by professional category is shown in Figure 1 (corresponding to question number 4).

The results recorded for question number 5 highlight the impact of age-related learning challenges among physicians, while financial and social status were more frequently cited by nurses.

(a) Pediatricians cited a love for children, their innocence, personal passion, and the complexity of the field as motivating factors.

(b) EPs emphasized the diversity, complexity, and challenges of the specialty, as well as passion for the field, the reward of making life-or-death decisions, its dynamic nature, and the need for continuous adaptability.

(c) ICU physicians also mentioned the diversity, complexity, and challenge of the specialty, along with the achievement of spectacular outcomes based on in-depth knowledge of the field.

(d) Nurses cited emotional involvement, empathy, and passion rooted in a desire to help patients; the aspiration to have a respected (respectable job) profession and financial considerations were also mentioned as reasons for choosing their career.

In response to question number 6, 414 (71.8%) responders answered affirmatively. The professional category that reported the highest level of managerial support for vocational training was nurses, while pediatric specialists reported the least support from their employers.

For question number 7, nurses represented the category with the greatest managerial support (79.7%), whereas pediatricians again reported the least support in this area.

Regarding involvement in working groups for developing workplace procedures, protocols, or guidelines (question number 8), EPs had the highest rate of non-participation (49.5%) compared to other professional categories included in the study.

Although 441 responders stated they were able to implement new knowledge acquired during scientific events in their workplace (question number 9), EPs had the highest rate of negative responses (28.1%), whereas nurses reported the highest affirmative response rate at 77.3%. In response to the questionnaires, 51.6% (298 responders) stated that their employer did not provide the necessary resources to manage tense or conflictual situations at work (question number 10). Among them, ICU-MDs and EPs made up the majority, at 63.7% and 61.7%, respectively (Table 1).

For question number 11, the responses were grouped based on the most frequent answers: 1. remuneration based on professional performance criteria; 2. difficult collaboration with colleagues, superiors, and staff from other specialties; 3. insufficient recognition, respect, and managerial support; 4. mental, verbal, and physical aggression of patients/family members; 5. insufficient procedural guidance and a defensive medical practice; 6. overwork/stress at work; and 7. limited availability of human resources and materials, as well as inadequate infrastructure. Of the total responders, 12.65% (9 pediatricians, 12 ICU-MDs, 12 EPs, and 40 nurses) did not answer this question, and 11.95% (4 pediatricians, 6 ICU-MDs, 15 EPs, and 44 nurses) believed there were no reasons for disappointment at work. The most common reasons for disappointment, as reported by the responders, included difficult collaboration with colleagues, superiors, and other specialists, disrespect, appreciation, and managerial support (indifference, disregard, and impartiality), overwork, and stress (large volume of duties). These responses were shared across the specialties participating in the study. Additionally, EPs reported, at a rate of approximately 28%, the practice of defensive medicine, the neglect of the specialty in terms of education, and the absence of work protocols as significant factors contributing to their disappointment at work (Table 2).

In total, 105 (18.19%) of the study participants (11 pediatricians, 14 ICU-MDs, 23 Eps, and 57 nurses) did not provide an answer to question number 12. The responses were grouped into the following categories: 1. remuneration based on professional performance criteria; 2. communication/debriefing courses; 3. managerial support, appreciation, and respect with the support of meritocracy; 4. education/vocational training courses; 5. implementation of protocols/procedures and application of sanctions; legislative change; 6. new premises/logistics (materials, equipment and medicines); 7. increasing the number of employees in all professional categories (human resources); 8. reorganization of the work schedule; and 9. restructuring of the sanitary system.

Grouping the responses recorded based on the most frequent suggestions revealed the following:

(a) Pediatricians—development and enforcement of procedures and protocols, communication courses and debriefing sessions, and staff education and participation in professional training courses;

(b) EPs—development and enforcement of procedures and protocols with their compliance and appropriate sanctions, new legislation regarding the restructuring of preventive primary medicine and the introduction of payments for non-emergencies, communication courses and debriefing sessions, staff education and participation in professional training courses, management restructuring with professionals in the field, and reorganization of the work schedules with allocation of senior medical personnel to work sectors with logistical attributions;

(c) ICU physicians—staff education and participation in professional training courses, communication courses and debriefing sessions, and salary remuneration based on performance criteria;

(d) Nurses—communication and professional training courses, improving infrastructure and logistics, increasing human resources and amending legislation to allow for retirement at the age of 57, and managerial support, including psychological counseling at work (Table 3).

The majority of responders represented from the two related medical specialties (emergency medicine, anesthesia and intensive care, respectively) supported the hypothesis of psycho-emotional and physical overload at work, with professional reorientation being an option for a significant percentage—26.0%—of EPs (Table 4).

The analysis of the independent variables revealed that age and years of experience in the profession were two factors that influenced selection for participation in groups dedicated to the development of medical guidelines/procedures/protocols, as well as the willingness to implement at work the novel knowledge acquired during scientific events. However, age and length of service were not identified criteria for choosing professional reorientation (Table S1).

When assessed as a whole, comparing the two professional categories nurses versus doctors, nurses reported the highest percentage of managerial support for professional training, access to updates in the field, involvement in working groups for the development of medical protocols/procedures, and support in overcoming workplace conflict. Doctors, on the other hand, perceived themselves as a managerially disadvantaged specialty, and they also reported the highest level of burnout at work (Table S2).

## 4. Discussion

This study aimed to address several complex areas among four distinct types of healthcare providers, such as motivations for choosing emergency medicine and job satisfaction, availability of organizational support, perception of malpractice risk, and organizational factors contributing to burnout.

### 4.1. Motivations for Choosing Emergency Medicine and Job Satisfaction

The issue of human resources in the field of emergency medicine is difficult to address and requires a multifactorial analysis.

The first step is represented by the motivational factors that influence students and young physicians to choose this specialty. Data from the literature highlight multiple factors, which can be classified into three categories: aspects related to clinical practice (focused on acute care, wide diversity of clinical conditions, inadequacy of long-term doctor–patient relationships, frontline physician role, adrenaline seekers); social aspects (emergency medicine as a lifestyle, personality fit, perceived salary, shift work, controlled working hours, flexible schedules, perceived prestige, level of risk and stress); and experiences during clinical rotations (professional role models, influence of senior physicians and mentors, length of residency) [8,9]. Under these conditions, students’ interest in a career in emergency medicine ranges from 10%, as reported by the American College of Emergency Physicians for the years 2005/2006 [10], to 6.1%, as reported in Canada for the years 2001–2004 [11].

The study results recorded regarding the reason for choosing the specialty (question number 5) highlighted aspects related to clinical practice for EPs (focused on acute care, wide diversity of clinical conditions, adrenaline junkies) as well as for ICU-MDs (complexity and challenge of the specialty, with the achievement of spectacular results) and pediatricians (complex field of specialization) working in emergency services. This contrasts with other studies, wherein social aspects (perceived salary, shift work, level of risk and stress) also play an important role due to their impact on well-being [8,9].

In the case of nurses, the aspects align with the data from the literature, highlighting social aspects such as financial considerations and the desire to increase prestige, in addition to clinical practice aspects related to improving knowledge and skills [12,13].

### 4.2. Availability of Organizational Support and Perception of Malpractice Risk

An important aspect that contributes to the choice of and retention within the specialty of emergency medicine is the opportunity to perform in a motivational environment (training, innovation, flexibility, remuneration, balanced between science and practice) through management involvement and the gurantee of logistical support in line with the needs and technological updates (achieving new standards and performance indicators) [14,15]. Conflict resolution mechanisms are also essential, as they help counterbalance the risk of malpractice by enhancing the quality and safety of medical care. Although the literature data highlight the role of management strategies in EDs, considered “shop window and a door to the hospital, difficult to control” [14], the evidence from the study shows that 28.2% of the responders did not benefit from training support (question number 6, Table 1), and 35.7% did not have access to the necessary resources to stay updated with developments in the field (question number 7, Table 1). This negatively impacts the quality of medical care and, implicitly, generates a sense of “frustration” among staff. According to management principles, these results should lead to the analysis of indicators for creating strategies [16] and should realign management directions, introducing protocols and clinical decision-support systems, based on evidence obtained through simulation, “process mining and role interaction models” in the EDs and performance “dashboard” analysis [14,16,17,18]. Regarding the presence of performance-enhancing tools in organizations, question number 8 highlights a major systemic issue, revealing that 41.8% of the responders were not involved in the development of protocols, procedures, or practice algorithms. This was predominantly seen among doctors, while the rate for pediatricians was 53.3%, that for emergency physicians was 49.5%, and for ICU-MDs, it was 47.5% (Table 1). However, it was noted that those who participated in training programs, at a significant rate of 76.4%, were able to apply the new knowledge in their practical work. All these findings related to deficiencies in the system’s offerings regarding performance development are reflected in the critical levels (over 30% of respondents globally and by category, Table 2) and represent reasons for disappointment in the workplace, with a potential impact on abandonment, as follows:

a. Insufficient recognition, respect, and managerial support across all professional categories (ranging from 34.3% of pediatricians to 46.5% of EPs), with possible behavioral changes or specialty shifts;

b. Overwork/stress in the workplace, particularly highlighted among EPs (42.01%) and ICU-MDs (35.48%), which can lead to exhaustion, burnout, and, consequently, to medical errors or malpractice. These issues have been further emphasized at the level of the Romanian healthcare system in studies conducted on emergency medical personnel in the intensive care team, in both pre-hospital and hospital settings [4,5].

Smith and colleagues highlight the role of leaders, managers, and senior professionals in inspiring, motivating, and developing the team in the emergency department [19]. This study’s findings on disappointment among senior doctors, who felt disadvantaged by management (Table S2), represent a vulnerable point in the system that may lead to decreased performance due to a “gradient of authority” and phenomena like the presence of “movers and shakers” in ED teams.

The literature data show that every ED faces communication problems and conflicts, most frequently at the interface with patients’ families or between specialties, which is why it is necessary to develop evaluation indicators and conflict management procedures structured via a conflict resolution framework that fosters collaboration [14,18,20]. Organizational or clinical practice communication deficits lead to interpersonal or interinstitutional conflicts that impact medical safety [20,21], patient safety, and sometimes even the safety of the emergency team. These represent social networking issues that require resolution through the development of communication tools [14,22].

This study also highlighted a systemic issue, namely, the employer’s inadequate role in resolving conflicts, reported globally by 51.6% of the responders, an aspect significantly noted by doctors across all specialties (Table 1). The lower percentage of non-involvement reported by nurses, only 38.7%, is due to the fact that they primarily experience conflicts within the emergency team, and they have minor responsibilities regarding communication with other departments or patient handover. Generally speaking, human resources management has had the primary goal of reducing staff instability, which requires avoiding employee dissatisfaction and improving decision-making processes by creating an operational model to reduce stress and burnout levels [14,15].

The responders in this study identified three major directions for improving work activity and increasing staff satisfaction:

a. Revising the employer’s management strategy, particularly regarding professional development for doctors;

b. Ensuring standardized communication tools and involvement in conflict resolution, potentially through team debriefing sessions (35.59%);

c. Introducing procedures, protocols, and algorithms, with practical implementation to meet healthcare needs (39.3% of EPs).

Nurses also raised issues related to equipment, finances, and psychological counseling. The evidence shows that even though, at the social level in Romania, the financial factor is identified as the primary reason for doctors leaving and choosing positions abroad, in reality, the respondents indicated organizational and systemic factors, such as training, team communication and case debriefing, managerial support for protocol implementation, and conflict resolution, as contributing factors, each reported at statistically significant levels of over 30%.

### 4.3. Organizational Factors Contributing to Burnout

Another important element described in the literature is the way the issues raised are reflected in the perception of stress and more pronounced deterioration among emergency and intensive care physicians. These professionals work with life-threatening emergencies, where the pressure of time, the severity of cases, insufficient protocol implementation, limited autonomy of staff, and inadequate patient flow lead to overcrowding, delays in diagnosis, inequities in care, excessive workload, and, consequently, to the onset of burnout phenomena [4,5]. Interfactorial analysis shows that poor training and communication, combined with limited managerial engagement, lead to negative effects in the workplace, causing disappointment, burnout, and a tendency toward professional reorientation. The percentage of ICU-MDs and EPs who would no longer choose their specialty is significantly high, at approximately 26%. It is worth noting that age and years of work experience were not factors that influenced the decision to change professions for these responders.

If we take into account the data highlighted in this study, which show that approximately 30% of employed EPs no longer wished to continue in this specialty (significant critical impact, *p* < 0.0001), along with the nationally quantified 30% shortage of emergency doctors, the collapse of emergency medicine appears to be a plausible projection at this time, raising serious red flags. Creating a culture of feedback and communication and developing training opportunities for both doctors and nurses, with leadership involvement and strategies tailored to the organization’s operational dashboard, can increase staff satisfaction and help prevent turnover and dropout [14,16,23,24].

Although an individual analysis of these complex areas was attempted, we observed numerous interconnections between the data presented, with interdependent processes and effects over time. This often necessitates a comprehensive approach, requiring us to view these elements as a cohesive whole.

This study’s limitations are related to the participation of responders working in the emergency departments of university hospitals, which have similar profiles in terms of case complexity, pressure from severe cases, and equipment. There are some differences based on the hospital type (monobloc vs. pavilion-style) and the motivational factors created by university educational offerings. These findings cannot be extrapolated to municipal or small-town EDs, where the interaction style, communication, social environment, and management strategies differ, thus potentially revelaing additional triggering factors with negative impacts on the emergency medical team. At the same time, considering the voluntary and self-administered nature of the questionnaire, we do not dispute the possibility of potential response and self-selection bias in the open-ended questions of the survey.

The practical implications of this study lie in the identification of systemic issues that reduce the performance of medical care, lead to professional dissatisfaction, and deteriorate the well-being of staff, resulting in abandonment. The absence of a strategic approach to the identified aspects creates a negative network that undermines healthcare delivery, a decrease in the quality and safety of medical practice, followed by abandonment among professionals (more pronounced among physicians), increased pressure on emergency teams with major risks of medical errors, burnout, and a return to higher staff turnover and professional reorientation.

This study suggests multiple directions for research in the organizational, educational, and psychological impact fields within emergency medical services. The goal is to identify predictive elements and create operational models to optimize leadership strategies, communication, and performance within the healthcare system.

## 5. Conclusions

This study constructed a “radiography” of the motivational, structural, management, and performance problems in EDs that have a major impact on specialized human resources and, implicitly, on the quality of the medical profession in the near future. Employer–professional relationship problems with critical values were highlighted, along with factors that may contribute to the re-consolidation of the management strategy to redefine the atmosphere of complexity, chaos, and dissatisfaction as one of teamwork, in harmony with patient and professional satisfaction, to ensure quality medical care in the ED.

This study highlights four paths for development, which represent the pillars of the reconsolidation of a prospective healthcare system focused on reducing risks by avoiding malpractice and increasing the quality of the medical act, with the main actors being human resources and patients. These include the development of procedures and protocols with uniform and non-discriminatory implementation; managerial reorganization (promotion of staff with managerial skills such as leadership, with appreciation and respect being essential here); the identification at the national level of new strategies to restore the trust of the medical staff in the system, with a reconsideration of the value system and academic development via professional training; and the prioritization of human resources, patient safety, appropriate endowment, the elimination of mediocrity, and the implementation of meritocracy.

## Figures and Tables

**Table 1:** Availability of organizational support for professional performance at work.

			ED Nurses	ICU Physicians	Emergency Physicians	Pediatric Physicians	Total Responders	*p*
Question 6	Yes	N (%)	213 (83.2%)	46 (57.5%)	131 (66.8%)	24 (53.3%)	414 (71.8%)	<0.001
	No	N (%)	43 (16.8%)	34 (42.5%)	65 (33.2%)	21 (46.7%)	163 (28.2%)	
Question 7	Yes	N (%)	204 (79.7%)	38 (47.5%)	113 (57.7%)	16 (35.6%)	371 (64.3%)	<0.001
	No	N (%)	52 (20.3%)	42 (52.5%)	83 (42.3%)	29 (64.4%)	206 (35.7%)	
Question 8	Yes	N (%)	174 (68.0%)	42 (52.5%)	99 (50.5%)	21 (46.7%)	336 (58.2%)	<0.001
	No	N (%)	82 (32.0%)	38 (47.5%)	97 (49.5%)	24 (53.3%)	241 (41.8%)	
Question 9	Yes	N (%)	198 (77.3%)	65 (81.3%)	141 (71.9%)	37 (82.2%)	441 (76.4%)	0.242
	No	N (%)	58 (22.7%)	15 (18.8%)	55 (28.1%)	8 (17.8%)	136 (23.6%)	
Question 10	Yes	N (%)	157 (61.3%)	29 (36.3%)	75 (38.3%)	18 (40.0%)	279 (48.4%)	<0.001
	No	N (%)	99 (38.7%)	51 (63.7%)	121 (61.7%)	27 (60.0%)	298 (51.6%)	

Legend: N = the number of responders.

**Table 2:** Reasons for disappointment at work among the responders.

Responses Categories		ED Nurses 172 (39.54%)	ICU Physicians 62 (14.25%)	Emergency Physicians 169 (38.85%)	Pediatric Physicians 32 (7.35%)	Total Responders 435 (100%)
1	N (%)	22 (12.79%)	8 (12.9%)	6 (3.55%)	0 (0%)	36 (8.27%)
2	N (%)	78 (45.34%)	25 (40.32%)	78 (46.15%)	11 (34.37%)	192 (44.13%)
3	N (%)	81 (47.09%)	25 (40.32%)	55 (32.54%)	12 (37.5%)	173 (39.77%)
4	N (%)	17 (9.88%)	5 (8.06%)	19 (11.24%)	3 (9.37%)	44 (10.11%)
5	N (%)	21 (12.20%)	14 (22.58%)	47 (27.81%)	6 (18.75%)	88 (20.22%)
6	N (%)	42 (24.41%)	22 (35.48%)	71 (42.01%)	9 (28.12%)	144 (33.10%)
7	N (%)	41 (23.83%)	17 (27.41%)	39 (23.07%)	2 (6.25%)	99 (22.75%)

Legend: color risk key, green (0–10%)—minor; yellow (>10–20%)—moderate; orange (>20–30%)—major; red (>30%)—critical; N = the number of responders.

**Table 3:** The responders’ suggestions for avoiding negative effects at work.

Response Categories		ED Nurses 199 (42.16%)	ICU Physicians 66 (13.98%)	Emergency Physicians 173 (36.65%)	Pediatric Physicians 34 (7.2%)	Total Responders 472 (100%)
1	N (%)	22 (11.05%)	18 (27.27%)	14 (8.09%)	2 (5.88%)	56 (11.86%)
2	N (%)	87 (43.71%)	20 (30.30%)	51 (29.47%)	10 (29.41)	168 (35.59%)
3	N (%)	21 (10.55%)	15 (22.72%)	32 (18.49%)	1 (2.94%)	69 (14.61%)
4	N (%)	19 (9.54%)	31 (46.96%)	44 (25.43%)	14 (41.17%)	108 (22.88)
5	N (%)	33 (16.58%)	11 (16.66%)	68 (39.30%)	11 (32.35)	123 (26.05%)
6	N (%)	35 (17.58%)	13 (19.69%)	40 (23.12%)	5 (14.7%)	93 (19.7%)
7	N (%)	40 (20.1%)	12 (18.18%)	39 (22,54%)	8 (23.52%)	99 (20.97%)
8	N (%)	11 (5.52%)	4 (6.06%)	12 (6.93%)	5 (14.7%)	32 (6.77%)
9	N (%)	3 (1.5%)	5 (7.57%)	6 (3.46%)	1 (2.94%)	15 (3.17%)

Legend: color risk key, green (0–10%)—minor; yellow (>10–20%)—moderate; orange (>20–30%)—major; red (>30%)—critical; N = the number of responders.

**Table 4:** Exhaustion and desire for professional reorientation by studied responders.

			ED Nurses	ICU Physicians	Emergency Physicians	Pediatric Physicians	Total Responders	*p*
Question 13	Yes	N (%)	211 (82.4%)	77 (96.3%)	183 (93.4%)	25 (55.6%)	496 (86.0%)	<0.001
	No	N (%)	45 (17.6%)	3 (3.8%)	13 (6.6%)	20 (44.4%)	81 (14.0%)	
Question 14	Yes	N (%)	212 (82.8%)	62 (77.5%)	145 (74.0%)	40 (88.9%)	459 (79.5%)	0.045
	No	N (%)	44 (17.2%)	18 (26.0%)	51 (26.0%)	5 (11.1%)	118 (20.5%)	

Legend: N = the number of responders.

## Data Availability

Upon reasonable request, the datasets from the current study will be made available by the corresponding author.

## Supplementary Materials

The following supporting information can be downloaded at: https://www.mdpi.com/article/10.3390/jcm14103316/s1, Figure S1: Study questionnaire; Table S1: Analysis of independent variables in the closed-ended questions; Table S2: Comparative analysis of the closed-ended questions.

## Abbreviations

The following abbreviations are used in this manuscript:

EPs	Emergency physicians
ED	Emergency department
ICU	Intensive care unit
ICU-MDs	Intensive care unit medical doctors
ASIDU	Association for Interdisciplinary Studies in the Field of Emergency Medicine

## References

[1] https://legislatie.just.ro/Public/DetaliiDocument/86455.

[2] https://insse.ro/cms/.

[3] https://romania.europalibera.org/a/deficit-de-medici-romania-ministerul-sanatatii-/32868819.html.

[4] Puticiu M., Grecu M.B., Rotaru L.T., Butoi M.A., Vancu G., Corlade-Andrei M., Cimpoesu D., Tat R.M., Golea A. (2024). Exploring Burnout, Work Addiction, and Stress-Related Growth among Prehospital Emergency Personnel. Behav. Sci..

[5] Butoi M.A., Vancu G., Marcu R.C., Hermenean A., Puticiu M., Rotaru L.T. (2025). The Role of Personality in Explaining Burnout, Work Addiction, and Stress-Related Growth in Prehospital Emergency Personnel. Healthcare.

[6] Bordianu C. Patient safety reflected in ethical management and patient rights. Proceedings of the Fifth Edition of the SIGMED International Conference, Titled “Patient Safety—A Goal of Medical Service Quality Management Systems”.

[7] https://www.viata-medicala.ro/malpraxisul-medical-in-romania-ce-trebuie-stiut-15838.

[8] Chew S.H., Ibrahim I., Yong Y.Z., Shi L.M., Zheng Q.S., Samarasekera D.D., Ooi S.B. (2018). Factors influencing the decision to pursue emergency medicine as a career among medical students in Singapore. Singap. Med. J..

[9] Rosen B., Rosen P., Schofer J., Asher S., Wald D., Cheaito M.A., Epter M., Kazzi A. (2019). Is Emergency Medicine the Right Choice for Me?. J. Emerg. Med..

[10] Boyd J.S., Clyne B., Reinert S.E., Zink B.J. (2009). Emergency medicine career choice: A profile of factors and influences from the Association of American Medical Colleges (AAMC) graduation questionnaires. Acad. Emerg. Med..

[11] Scott I.M., Abu-Laban R.B., Gowans M.C., Wright B.J., Brenneis F.R. (2009). Emergency medicine as a career choice: A descriptive study of Canadian medical students. Can. J. Emerg. Med..

[12] Alsalah A.Y., Alkarani A.S. (2023). Exploring the motivation and barriers that nurses experience when enrolling for a Master’s in emergency and disaster nursing. Nurs. Commun..

[13] Cevik A.A., Cakal E.D., Shaban S., El Zubeir M., Abu-Zidan F.M. (2021). A mandatory Emergency Medicine clerkship influences students’ career choices in a developing system. Afr. J. Emerg. Med..

[14] Seow E. (2013). Leading and managing an emergency department-A personal view. J. Acute Med..

[15] Mahdavi A., Atlasi R., Ebrahimi M., Azimian E., Naemi R. (2023). Human resource management (HRM) strategies of medical staff during the COVID-19 pandemic. Heliyon.

[16] Mostafa R., El-Atawi K. (2024). Strategies to Measure and Improve Emergency Department Performance: A Review. Cureus.

[17] Alvarez C., Rojas E., Arias M., Munoz-Gama J., Sepúlveda M., Herskovic V., Capurro D. (2018). Discovering role interaction models in the Emergency Room using Process Mining. J. Biomed. Inform..

[18] Catherine E. (2017). Perron, Richard G. Bachur, Anne M. Stack. Development, Implementation, and Use of an Emergency Physician Performance Dashboard. Clin. Pediatr. Emerg. Med..

[19] Burton N. (2004). Leading the Professionals: How to inspire & motivate professional service teams [Book Review]. Eng. Manag. J..

[20] Creswick N., Westbrook J.I., Braithwaite J. (2009). Understanding communication networks in the emergency department. BMC Health Serv. Res..

[21] Counselman F.L., Schafermeyer R.W., Garcia R., Perina D.G. (2000). A survey of academic departments of emergency medicine regarding operation and clinical practice. Ann. Emerg. Med..

[22] Eisenberg E.M., Murphy A.G., Sutcliffe K., Wears R., Schenkel S., Perry S., Vanderhoef M. (2005). Communication in Emergency Medicine: Implications for Patient Safety. Commun. Monogr..

[23] Patel R.S., Sekhri S., Bhimanadham N.N., Imran S., Hossain S. (2019). A Review on Strategies to Manage Physician Burnout. Cureus.

[24] Aguirre R.R., Suarez O., Fuentes M., Sanchez-Gonzalez M.A. (2019). Electronic Health Record Implementation: A Review of Resources and Tools. Cureus.

