# Supplementary material for: Thirty Years of Emergency Medicine in Romania—A Bridge Between the Behavior of Emergency Department Professionals and the Health System Management Strategy: A Survey Study
Source: J Clin Med. 2025 May 9;14(10):3316. doi: 10.3390/jcm14103316 (PMC12112167; doi:10.3390/jcm14103316)
Supplement: Supplementary file 1 [file jcm-14-03316-s001.zip › jcm-3623268-supplementary.pdf]

Supplementary Materials

1. Sex F M
2. Age (Years) ……………………
3. Respondents ' professional seniority
4. Specialty ……………………………………………
5. What was the reason why you chose this specialty?

   ………………………………………………………………………………………
6. During your activity, did you receive support from the employer for professional training courses?

   Yes No
7. During the period of your activity, did the employer provide you with the necessary means to access updates in your specialty?

   Yes No
8. Have you participated in a working group dedicated to the development of workplace guidelines/protocols/procedures?

   Yes No
9. Were you able to implement at work the new knowledge acquired during scientific events?

   Yes No
10. Did the employer provide you with the necessary resources to handle tense/conflicting situations at work?

    Yes No
11. What are the main reasons why you feel disappointed at work?

    ………………………………………………………………………………………
12. What do you suggest could be improved at the workplace to increase satisfaction/efficiency?

    ………………………………………………………………………………………
13. Do you consider your chosen specialty to be more stressful/consumptive than other specialties?

    Yes No
14. If you had to do it all over again, would you still choose this profession/specialty?

    Yes No

Figure S1. Study questionnaire.

Table S1. Analysis of independent variables in the closed-ended questions.

Questions	Analyzed variables (years)	YES (mean ± standard deviation)	NO (mean ± standard deviation)	p
Question 6	Age	40±9.3	41.5±9.5	0.09
	Length of service	13.6±10	15.1±9.2	0.08
Question 7	Age	40±9.2	41.1±9.7	0.1
	Length of service	13.7±9.8	14,6±9.7	0.2
Question 8	Age	41,6±9.2	38,7±9.4	< 0.001
	Length of service	15,3±9.8	12,2±9.4	< 0.001
Question 9	Age	41.1±9.5	38,4±8.9	0.004
	Length of service ience	14.7±9.9	11.8±9.1	0.003
Question 10	Age	40.2±9.1	40.6±9.7	0.6
	Length of service	13.8±9.8	14.2±9.8	0.5
Question 13	Age	40.3±9.6	42±8.7	0.1
	Length of service	14.1±9.9	14.1±9.3	0.9

Question 14	Age	40,7±9.5	39,9±9.7	0.4
	Length of service	14±9.8	14.4±9.7	0.6

Legend: Legend: N = the number of responders.

Table S2. Comparative analysis of the closed-ended questions.

			ED nurses	Physicians	Total responders	p
Question 6	Yes	N (%)	213 (83.2%)	199 (62.6%)	412 (71.8%)	< 0.001
	No	N (%)	43 (16.8%)	119 (37.4%)	162 (28.2%)	
Question 7	Yes	N (%)	204 (79.7%)	166 (52.2%)	370 (64.5%)	< 0.001
	No	N (%)	52 (20.3%)	152 (47.8%)	204 (35.5%)	
Question 8	Yes	N (%)	174 (68.0%)	159 (50.0%)	333 (58.0%)	< 0.001
	No	N (%)	82 (32.0%)	159 (50.0%)	241 (42.0%)	
Question 9	Yes	N (%)	198 (77.3%)	240 (75.5%)	438 (76.3%)	0.6
	No	N (%)	58 (22.7%)	78 (24.5%)	136 (23.7%)	
Question 10	Yes	N (%)	157 (61.3)	122 (38.4%)	279 (48.6%)	< 0.001
	No	N (%)	99 (38,7%)	196 (61.6%)	295 (51.4%)	
Question 13	Yes	N (%)	211 (82.4%)	285 (88.8%)	496 (86.0%)	0.03
	No	N (%)	45 (17.6%)	36 (11.2%)	81 (14.0%)	
Question 14	Yes	N (%)	212 (82.8%)	247 (76.9%)	459 (79.5%)	0.1
	No	N (%)	44 (17.2%)	74 (23.1%)	118 (20.5%)	

Legend: Legend: N = the number of responders.
